# Ventricular arrhythmias originating from left ventricular summit: Salvage strategies after conventional ablation

**DOI:** 10.1002/joa3.12991

**Published:** 2024-01-11

**Authors:** Dat Cao Tran, Fa‐Po Chung

**Affiliations:** ^1^ Heart Rhythm Center, Division of Cardiology, Department of Medicine Taipei Veterans General Hospital Taipei Taiwan; ^2^ Department of Medicine National Yang‐Ming Chiao‐Tung University School of Medicine Taipei Taiwan

Ventricular arrhythmias (VAs) originating from the left ventricular (LV) summit account for 10%–12% of idiopathic VAs. Catheter ablation of LV summit VA, especially for intramural origins, poses a clinical challenge. Given the anatomical complexity of the LV summit, adjacent structures such as aortic sinus cusps, the subvalvular region of the left ventricular outflow tract (LVOT), epicardial aspects from the great cardiac vein/anterior interventricular vein (GCV/AIC), or the right ventricular outflow tract (RVOT) may serve as vantage points to achieve successful ablation. Despite the heterogeneous initial approach locations, failed ablation or recurrences of LV summit VAs are often attributed to inadequate energy delivery/penetration or inappropriate localization.^1^ Therefore, several bailout strategies, such as simultaneous unipolar ablation, bipolar ablation, half‐saline irrigant ablation, ethanol injection, needle ablation, or pulsed‐field ablation, have been proposed to improve clinical outcomes.

In the case reported by Sudo et al., a bipolar ablation approach was used as a bailout therapy after ineffective endocardial attempts at the RVOT and LVOT. The early signal documented in an epicardial branch of the coronary sinus, along with a high correlation during pacing, suggested an epicardial origin of the PVC or PVC exit from epicardium. Through applying bipolar ablation between the epicardial portion of the LV summit and the LV endocardium, successful PVC elimination was achieved. Bipolar ablation has demonstrated efficacy in treating deep‐seated arrhythmias, especially for those that are transmural. Different placements of ablation catheters for bipolar ablation should also be considered, such as supravalvular and subvalvular regions of LVOT, or RVOT and LVOT.^2^ Detailed mapping of the surrounding anatomic structures is essential for clinical decision‐making for the location of ablation catheters for the bipolar approach. Additionally, bipolar ablation could also be applied to different structures with catheters surrounding the arrhythmia origin, enabling energy to travel across catheters and targets.

While acute success was achieved using bipolar ablation, it is important to recognize the potential risks and failures associated with this approach. Locating the exact positions for the two polarities can be challenging. This strategy may involve trial and error. Energy delivery in this manner may transiently suppress PVCs due to the extensive lesions created, but recurrences may ensue during follow‐up as the acute effects wane. An improperly created transmural scar from prolonged ablation could pose risks, such as steam pops. Additionally, inaccessible regions of the LV summit, particularly due to adjacent coronary vessels that are vulnerable to RF energy application, can complicate the application of bipolar ablation.

Careful energy titration, consideration of adjacent coronary courses, and demonstrated anatomical understanding, as exemplified in the reported case, are imperative. Despite achieving acute success, late recurrences may occur, potentially influenced by edematous tissues created during prior endocardial ablation and hindered transmural effects of bipolar ablation. The choice of catheter tip orientation (parallel vs. perpendicular) and the use of an 8‐mm catheter for the returning electrode are factors that can impact energy delivery and deserve consideration.^3^

Alternative strategies for LV summit VAs that are refractory to conventional approaches include multi‐site ablation, which has shown efficacy in eliminating PVCs but would be associated with higher recurrences. The acute success rate was similar when approaching the surrounding anatomic structures, such as aortic cusps, subvalvular regions, or GCV/AIV, highlighting the critical role of mapping adjacent structures as the vantage point to achieve successful ablation. Prolonging ablation duration and increasing energy settings to a safe extent before resorting to more invasive methods may be justified for deep arrhythmogenic foci. The use of half‐normal saline as an irrigant could create deeper lesions might be a viable option for LV summit VA, though higher recurrences had been reported.

Intriguingly, the insertion of a 2Fr microcatheter with early signal documentation in the presented case may provide a clue for the consideration of retrograde coronary venous ethanol ablation (RCVEA). This technique, deemed safe for LV summit ablation, has shown high acute success rates and reasonable freedom from recurrence during follow‐up.^4^

As a novel energy source, pulse‐field ablation has emerged with promising potential for creating deeper lesions while sparing adjacent structures. Preliminary animal studies have demonstrated lesion formation up to 10 mm in depth.^5^

In conclusion, bipolar ablation stands as an effective treatment for transmural VAs, as highlighted in the report by Sudo et al. With careful preparation, a nuanced understanding of outflow tract anatomy, and strategic deployment, bipolar ablation can serve as a bailout strategy for PVCs/VT refractory to conventional endocardial approaches.

## CONFLICT OF INTEREST STATEMENT

Authors declare no conflict of interests for this article.
